# Intracellular bactericidal activity and action mechanism of MDP1 antimicrobial peptide against VRSA and MRSA in human endothelial cells

**DOI:** 10.3389/fmicb.2024.1416995

**Published:** 2024-08-26

**Authors:** Shirin Dashtbin, Shabnam Razavi, Mokhtar Ganjali Koli, Farnoosh Barneh, Sarvenaz Ekhtiari-Sadegh, Reza Akbari, Gholamreza Irajian, Kamran Pooshang Bagheri

**Affiliations:** ^1^Microbial Biotechnology Research Center, Iran University of Medical Sciences, Tehran, Iran; ^2^Department of Microbiology, Faculty of Medicine, Iran University of Medical Sciences, Tehran, Iran; ^3^Department of Chemistry, University of Kurdistan, Sanandaj, Iran; ^4^Computational Chemistry Laboratory, Kask Afrand Exire Ltd., Sanandaj, Iran; ^5^Venom and Biotherapeutics Molecules Laboratory, Medical Biotechnology Department, Biotechnology Research Center, Pasteur Institute of Iran, Tehran, Iran; ^6^Department of Microbiology and Virology, School of Medicine, Urmia University of Medical Sciences, Urmia, Iran

**Keywords:** intracellular *S. aureus*, antimicrobial peptide, melittin-derived peptide, VRSA, MRSA, endothelial cell

## Abstract

**Introduction:**

*Staphylococcus aureus* is a prominent cause of postoperative infections, often persisting within host cells, leading to chronic infections. Conventional antibiotics struggle to eliminate intracellular *S. aureus* due to poor cell penetration. Antimicrobial peptides are a new hope for tackling intracellular bacteria. Accordingly, this study examines the antimicrobial peptide MDP1, derived from melittin, for its efficacy against intracellular *S. aureus*.

**Methods:**

In this study, the physiochemical properties (Prediction of three-dimensional structure, circular dichroism and helical wheel projection analysis) were investigated. Extracellular antibacterial activity and cytotoxicity of MDP1 were also assessed. The mechanism of interaction of MDP1 with *S. aureus* was evaluated by molecular dynamic simulation, atomic force and confocal microscopy. Bacterial internalization into an endothelial cell model was confirmed through culture and transmission electron microscopy. The effect of the peptide on intracellular bacteria was investigated by culture and epi-fluorescence microscopy.

**Results and discussion:**

3D structural prediction proved the conformation of MDP1 as an α-helix peptide. Helical-wheel projection analysis indicated the proper orientation of hydrophobic amino acid residues for membrane interaction. CD spectroscopy of MDP1 showed that MDP1 in SDS 10 and 30 mM adopted 87 and 91% helical conformation. Atomic force and confocal microscopy assessments as well as molecular dynamics studies revealed the peptide-bacterial membrane interaction. MDP1, at the concentration of 0.32 μg mL^−1^, demonstrated a fold reduction of 21.7 ± 1.8, 1.7 ± 0.2, and 7.3 ± 0.8 in intracellular bacterial load for ATCC, VRSA, and MRSA, respectively. Molecular dynamics results demonstrate a preferential interaction of MDP1 with POPG/POPE membranes, primarily driven by electrostatic forces and hydrogen bonding. In POPC systems, two out of four MDP1 interacted effectively, while all four MDP1 engaged with POPG/POPE membranes. Gathering all data together, MDP1 is efficacious in the reduction of intracellular VRSA and MRSA proved by culture and epi-fluorescent microscopy although further studies should be performed to increase the intracellular activity of MDP1.

## 1 Introduction

Globally, *Staphylococcus aureus* poses a significant challenge as one of the most prevalent opportunistic human pathogens, frequently causing recurrent bacterial infections (Noore et al., 2013). A critical aspect of its pathogenesis involves its ability to adopt an intracellular lifestyle, allowing the bacterium to evade antibiotic effects and host immunity (Soe et al., 2021). An invasion of host cells by *S. aureus* can lead to two main outcomes: (i) rapid host cell lysis, induced by the secretion of toxins and pro-inflammatory factors, resulting in significant inflammation and cytotoxicity; (ii) persistence within morphologically intact host cells for extended periods, when virulence factors are not secreted or downregulated (Rollin et al., 2017). These persisting bacteria, often referred to as “small colony variants” (SCVs), are associated with chronic infections, as initially described by Proctor et al. (1995). Recent research indicates that impaired growth of *S. aureus* SCVs is linked to altered metabolic activity (Amato et al., 2014; Wong Fok Lung and Prince, 2020). Furthermore, the SCV phenotype extends to other pathogenic bacterial species, highlighting its significance in persistent infections (Kahl et al., 2016).

*In vitro* studies demonstrate that *S. aureus* can intracellularly survive in various cell types, including epithelial cells, endothelial cells, osteoclasts, keratinocytes, and fibroblasts. However, the specific infectious cycle depends on the type of infected cells (Hommes and Surewaard, 2022). Generally, when invading non-specialized phagocytes, *S. aureus* utilizes a zipper-like mechanism involving fibronectin-binding proteins A and B (FnBPA and FnBPB). The interaction between these proteins and cell-associated fibronectin, as well as integrin α5β1, facilitates staphylococcal internalization within non-specialized phagocytes (Peacock et al., 1999; Foster et al., 2014). Initial adhesion to host cells is facilitated by α5β1 integrins and FnBPs, leading to the development of phagocytic cups and bacterial endocytosis. Bacterial internalization through a process orchestrated by host cells involves actin remodeling, focal adhesion kinase (FAK), and Src family kinases (SFKs) (Agerer et al., 2005; Schröder et al., 2006). SCVs, characterized by elevated levels of FnBPs, exhibit enhanced invasion capabilities into host cells (Vaudaux et al., 2002). Upon internalization, *S. aureus* can encounter diverse intracellular fates: being disinfected within phagolysosomes, surviving within endosomes, or escaping into the host cell cytoplasm via mechanisms involving α-toxin, phenol-soluble modulins (PSM), and phospholipases (Fraunholz and Sinha, 2012).

The destiny of the pathogen and the infected host cell is contingent upon the staphylococcal isolate, genotype, and the variable susceptibility of host cells to virulence factors, as well as host cell gene expression (Missiakas and Winstel, 2020).

The intracellular aspect of *S. aureus* infections certainly impedes antibiotic efficiency and may even assist in acquiring antibiotic tolerance (Holmes et al., 1966). At their therapeutic dose, conventional antibiotics usually present low permeability, rendering the intracellular bacterial infection treatment ineffective (Buccini et al., 2020). Intracellular infections pose a challenge for traditional antibiotics, with only a limited number of new antimicrobials such as quinolones, clindamycin, and telavancin proving effective (Huo et al., 2020). Hence, the quest for novel therapies, like antimicrobial peptides (AMPs) against this adaptable pathogen is pressing (Bravo-Santano et al., 2018). AMPs are peptides characterized by their ability to target cell membranes, exhibiting different physical and chemical properties. They are known for their capacity to disrupt or destabilize cell membranes, leading to pore formation (Aghazadeh et al., 2020). Various factors affect this membrane-associated mechanism, such as steric effects between lipids, membrane curvature, and the differences in the number of adjacent molecules within the membrane (Cardoso et al., 2024).

Some AMPs like lactoferricin, tachyplesin, mastoparan, magainin, cecropin, gramicidin S, and melittin have demonstrated cytotoxic effects on eukaryotic cell membranes due to their membrane-disrupting nature, limiting their further development (Giangaspero et al., 2001). To overcome this major concern, some studies have focused on designing new peptides with reduced toxicity (Akbari et al., 2018; Memariani et al., 2018). Melittin is one of the potent AMPs but its high toxicity hinders its clinical applications. Akbari et al. (2018, 2022) tried to reduce the toxicity of melittin by some deletion in the peptide sequence. These efforts led to the development of MDP1 and MDP2 (melittin-derived peptides 1 and 2). The toxicity of MDP1 was lower than MDP2 (Akbari et al., 2018, 2022) and selected for study of its intracellular activity in this study. Analysis of the amino acid sequence of melittin identified two main hydrophobic motifs, namely, the N-terminal GIGAVLKVL and central GLPALISWI motifs. Two amino acid residues (W19 and I20) were deleted from the second hydrophobic motif. Additionally, S18 was removed to shorten the peptide (MDP1). Furthermore, its mode of action was investigated using various assays, including nucleic acid release assays, fluorescence release, and adsorption assays, and scanning electron microscopy (Akbari et al., 2018). Akbari et al. (2018) assessed the kinetics of antimicrobial activity, toxicity, and stability of MDP1.

Although MDP1’s antibacterial properties are known, it is unknown whether MDP1 is effective at killing intracellular *S. aureus* within the cells. Thus, the present study was designed to test this hypothesis that MDP1 is effective in killing intracellular *S. aureus*.

## 2 Materials and methods

### 2.1 Reagents, media, bacteria, and cells

Vancomycin, gentamicin, trypsin, sodium dodecyl sulfate (SDS), 3-[4,5-dimethylthiazol-2-yl]-2,5 diphenyl tetrazolium bromide (MTT), and Pen-Strep (penicillin–streptomycin) were purchased from Sigma-Aldrich Chemie Co. (Sigma-Aldrich Chemie GmbH, Taufkirchen, Germany). Mueller-Hinton Broth (MHB) and Mueller-Hinton Agar (MHA) for bacterial cultures were obtained from Merck (Darmstadt, Germany). Dulbecco’s Modified Eagle Medium (DMEM) and Fetal Bovine Serum (FBS) were sourced from Gibco, Life Technologies (Grand Island, NY, United States). The Smart BCA (Bicinchoninic acid assay) kit was acquired from Intron Biotechnology Co. (South Korea). RNA extraction solution (RNX) was obtained from SinaClon Co. (Iran). The cDNA synthesis kit was purchased from Favorgen Co. (Taiwan).

American Type Culture Collection (ATCC) 29,213, methicillin-resistant *S. aureus* (MRSA) and vancomycin-resistant *S. aureus* (VRSA) strains were previously isolated from Shahid Motahhari Burn Hospital (Bevalian et al., 2021). The cell lines used in this study were obtained from the Department of Cell Bank, Pasteur Institute of Iran. All experiments were conducted in triplicate and results are presented as mean ± standard deviation (SD).

### 2.2 Physiochemical properties

Hydrophobicity, Grand average of Hydropathicity (Gravy), net charge, isoelectric pH, Bouman index and molecular weight of the peptide were determined by ‘Protparam’ server^1^ (Gasteiger et al., 2005), Antimicrobial Peptide Calculator and Predictor; APD3 server^2^ at the University of Nebraska (Wang et al., 2016).

### 2.3 Prediction of three-dimensional structure

The 3D structure of MDP1 was predicted using the I-TASSER online server (Iterative Threading Assembly Refinement) at Michigan University^3^ (Roy et al., 2010). From the predicted structures, the peptide with the lowest root mean square deviation (RMSD) and the highest C-score (confidence score) was chosen. The predicted structure was visualized by the Chimera X software package (ver 1.6.1) (Pettersen et al., 2021). The integrity of predicted structures was confirmed by MolProbity analyses including rotamers, Ramachandran favored, Rama distribution *Z*-score, MolProbity score, and Cβ deviations^4^ (Lovell et al., 2003; Williams et al., 2018).

### 2.4 Simulation details

**Figure 1:**
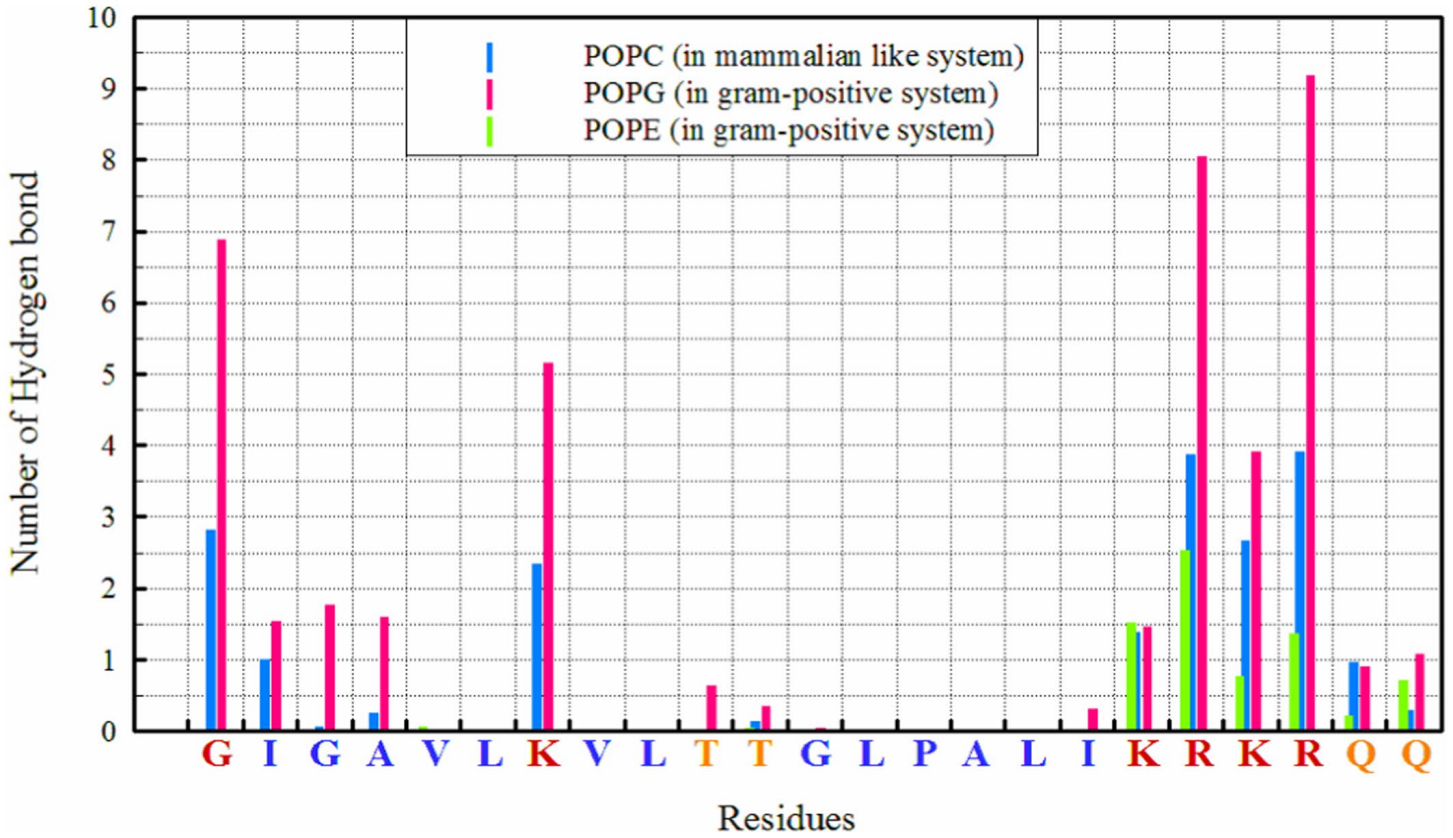
Number of hydrogen bonds between different residues and lipids in simulated systems.

All-atom MD simulations were performed for MDP1 in two phospholipid bilayer systems. One of the bilayer systems was modeled with a 3:1 mixture of palmitoyl oleoyl phosphatidylglycerol (POPG) and palmitoyl oleoyl phosphatidylethanolamine (POPE), mimicking the components of Gram-positive (GP) bacteria. The other system consisted of pure POPC lipids, mimicking the membrane components of mammalian-like (ML) membrane (Lee et al., 2016b; Balatti et al., 2018) Peptide-membrane systems for different simulations were constructed using CHARMM-GUI (Jo et al., 2009; Wu et al., 2014; Lee et al., 2016a) to produce symmetric bilayers with 75 POPG and 25 POPE lipids per leaflet for the GP membrane and 100 POPC lipids per leaflet for the ML membrane. For each system, 4 MDP1 molecules were placed ~1.2 nm above the bilayer surface initially, as seen in Figure 1. Sufficient amounts of sodium and chloride ions were added to each system to reach charge neutrality and achieve a salt concentration of 0.15 M. The simulations were executed using the GROMACS 2021.5 package (Lindahl et al., 2022a,b). The CHARMM36 force field (Klauda et al., 2010; Vanommeslaeghe et al., 2010) was applied to the peptide and all model membranes. All simulations were carried out at a temperature of 310 K and a pressure of 1 bar. The temperature was controlled by the Nose-Hoover thermostat (Nosé, 1984; Hoover, 1985) with a coupling time of 0.5 ps. The pressure was controlled by coupling the simulation cell to a Parrinello-Rahman barostat (Parrinello and Rahman, 1981), with a coupling time constant of 2 ps. Semi-isotropic pressure coupling was applied with two degrees of freedom, one in the xy direction and another in the z-direction.

In all simulation systems, periodic boundary conditions were used along all simulation box axes, and the transferable intermolecular potential 3-point (TIP3P) water model (Jorgensen et al., 1983) was applied to solve the systems. All-atom bond lengths were constrained using the LINCS algorithm (Hess, 2008). Newton’s equations of motion were integrated using the leap-frog algorithm (Hockney et al., 1974; Cuendet and Van Gunsteren, 2007) with a time step of 2 fs. Electrostatic and van der Waals (vdW) interactions were cut off at 1.2 nm, while long-range electrostatic interactions were treated using the particle mesh Ewald method (Darden et al., 1993; Essmann et al., 1995). In all systems, unfavorable atomic contacts were removed by the steepest descent energy minimization. Initially, while the positions of peptides were restrained, equilibration was conducted in the NVT ensemble for 1 ns, followed by equilibration in the NPT ensemble for 9 ns. After the equilibration steps, all simulations were run in the NPT ensemble for 500 ns from their starting conditions and coordinates of the atoms. All results were obtained from the last 10% of the simulation time.

### 2.5 Peptide synthesis

A synthetic and purified MDP1 (GIGAVLKVLTTGLPALIKRKRQQ) was manufactured by ChinaPeptides Co. (Shanghai, China). The peptide exhibited purity exceeding 97% as determined by reverse-phase high-performance liquid chromatography (RP-HPLC). The molecular masses of this peptide were verified using Electrospray Ionization Mass Spectrometry (ESI-MS) analysis. C-terminal amidation was carried out on the peptide, and its concentration was reassessed using a bicinchoninic acid assay kit. The data is presented in Supplementary Figures S1, S2.

### 2.6 Antimicrobial assay

Microdilution assay was performed to determine the minimal inhibitory concentration (MIC) and minimal bactericidal concentration (MBC) of MDP1. McFarland standard bacterial suspension (0.5 McFarland) was prepared by spectrophotometry at 625 nm. The number of bacteria in 0.5 McFarland suspension is equal to 1.5 × 10^8^ CFU mL^−1^ when the optical density at 625 nm is ranged from 0.08 to 0.1. In our study, the optical density (OD) of the suspension was set to the mid-point of 0.09 to increase the accuracy of bacterial quantification.

Briefly, MDP1 was serially diluted in a 96-well polypropylene microplate from 64 to 0.062 μg mL^−1^. 1.5 × 10^5^ CFU mL^−1^ bacteria were then added to each well, and incubated at 37°C for 24 h.

### 2.7 Circular dichroism spectroscopy

The secondary structure of MDP1 was studied by circular dichroism (CD) spectroscopy using an AVIV MODEL 215 spectropolarimeter (AVIV Instruments, Inc., Lakewood, NJ, United States). Initially, the peptide (0.25 μg mL^−1^) was resuspended in SDS (10 and 30 mM) to mimic a membrane environment (Zhang et al., 2016).

The samples were placed in 0.1 cm path length cuvettes (Hellma, Forest Hills, NY, United States) at room temperature. The spectra were recorded over a wavelength range of 190–260 nm at a scan speed of 350 cps at room temperature, with an average of five scans. The helical content of the peptide was calculated using CDNN 2.0 software (Dr. Gerald Bohm, Martin-Luther-University at Halle-Wittenberg, Germany).

### 2.8 Selection of the most pathogenic *S. aureus* by real-time PCR

*Staphylococcus aureus* possesses a diverse array of virulence factors, many of which are surface proteins covalently anchored to peptidoglycan (Foster et al., 2014). FnBPA and FnBPB stand out as highly adhesive proteins in *S. aureus*. Research indicates that the most pathogenic strains of *S. aureus* express higher levels of FnBPs (Menzies, 2003). FnBPs have been implicated in the invasion of various non-phagocytic cell lines (Speziale and Pietrocola, 2020). To identify the most pathogenic clinical strains, the expression of the *fnbpA* gene was assessed using real-time PCR.

The bacteria were cultured in MHB and incubated at 37°C to promote growth. Upon reaching the desired growth phase (mid-log phase for RNA isolation), the bacteria were harvested by centrifugation to separate them from the growth medium. Subsequently, the harvested bacteria underwent washing steps to eliminate any remaining growth medium or contaminants. Finally, the bacterial concentration was adjusted to match a 0.5 McFarland standard.

Total RNA was extracted using RNX reagent (Sinaclon, Iran), and the purity of RNA was evaluated by measuring the absorbance ratio at 260 nm and 280 nm, with a ratio of approximately 2.0 indicating pure RNA. Subsequently, cDNA was synthesized using the PrimeScript RT kit (Favorgen, Taiwan) following the manufacturer’s protocols. Quantitative Real-time polymerase chain reaction was performed using SYBR Green Master Mix to analyze the expression of *fnbpA*, employing Real-Time PCR System (Applied Biosystems, United States). The expression level of the housekeeping gene *16S rRNA* was utilized as an internal control to normalize the results. The primer sequences employed for the Real-time PCR analysis are detailed in Table 1.

**Table 1:** The primers used for Real-time PCR.

Genes	Sequences (5′–3′)		Tm (°C)	Product length (bp)	Reference
*fnbpA*	F	AAATTGGGAGCAGCATCAGT	60	121	Atshan et al. (2013)
	R	GCAGCTGAATTCCCATTTTC	60		
*16srRNA*	F	TCGTGTCGTGAGATGTTGGGTTA	58	195	Zhang et al. (2021)
	R	GGTTTCGCTGCCCTTTGTATTGT	58		

### 2.9 Evaluation of MDP1 toxicity on EA.hy 926 cells

*Staphylococcus aureus* colonizes cell surfaces but recent work has demonstrated that cell invasion of non-professional phagocytes essentially contributes to the development of infections (Strobel et al., 2016). We focused on endothelial cells, which have been previously shown to readily internalize staphylococci *in vitro* (Rollin et al., 2017).

The EA.hy 926 cell line (ATCC^®^ CRL-2922™), originally derived from a human umbilical vein, was used. The EA.hy 926 cells were grown in DMEM containing 10% FBS. The cells were seeded into DMEM media at the density of 4 × 10^4^ cells/well in a sterile 96-well plate for 24 h and then incubated with different concentrations of MDP1 (10.24–0.04 μg mL^−1^) for another 24 h. Untreated cells were used as the negative control.

MTT assay was performed to evaluate the viability of the cells. Briefly, 100 μL of the MTT reagent (final concentration 0.5 mg mL^−1^) was added to each well and incubated for 3 h in a humidified atmosphere (37°C, 5% CO_2_). The supernatant was removed, 100 μL of isopropyl alcohol was added to each well, and the plate was incubated with shaking at 37°C for 30 min. The absorbance was then measured at 570 nm. The assay was performed independently in triplicate. The percentage of viability was calculated using the following formula:


$$\mathrm{Viability}\%=\frac{{\mathrm{OD}}_{\mathrm{sample}}-{\mathrm{OD}}_{\mathrm{negative control}}}{{\mathrm{OD}}_{\mathrm{control}}-{\mathrm{OD}}_{\mathrm{negative control}}}\times 100$$

### 2.10 *In vitro* peptide-membrane interaction

#### 2.10.1 Effect of fluorescein isothiocyanate (FITC)-labeled MDP1 on *S. aureus* membrane

*Staphylococcus aureus* in its mid-logarithmic growth phase was diluted to a concentration of 10^7^ CFU mL^−1^ using PBS (pH 7.4). The bacterial suspension was then co-incubated with a FITC-labeled MDP1 at 0.32 μg mL^−1^ (non-toxic concentration) for 1 h. After incubation, the cells were washed three times with PBS and visualized using a confocal laser scanning microscope (CLSM) (Leica TCS SPE, America) (He et al., 2020).

#### 2.10.2 Atomic force microscopy (AFM)

The morphological alterations of MRSA and VRSA after incubation with MDP1 were further examined using AFM. In brief, mid-log phase bacterial cultures (1 × 10^7^ CFU mL^−1^) were exposed to 0.32 μg mL^−1^ of peptides at 37°C for 1 h. After washing three times with distilled water, a 20 μL bacterial suspension was placed on mica and incubated for 30 min at room temperature (Ji et al., 2023). A non-contact cantilever tip with a spring constant of 200 N/m and a resonance frequency of 90 kHz was used. A scan speed of 1.5 Hz was set and resulted in a final resolution of 512 by 512 pixels.

### 2.11 Evaluation of *S. aureus* internalization

This assay was conducted to address the following objectives:

- To confirm the internalization of *S. aureus* bacteria into the cells.
- To demonstrate that the internalized bacteria persist within the cells.

To achieve this, the multiplicity of infection (MOI) was determined to find the optimal number of bacteria. Internalization was validated through bacterial culture, and the internalized bacteria were observed using Transmission Electron Microscopy (TEM).

#### 2.11.1 Determination of MOI

The MOI refers to the number of the bacterial particle(s) present relative to the host cell(s). At the optimal bacteria-to-cells ratio, the number of internalized bacteria is maximized.

At this stage, the method employed by Siegmund et al. (2021) was utilized with some modifications.

EA.hy926 cells were seeded in a 24-well plate. The bacteria were grown in MHB at 37°C for 24 h, harvested by centrifugation at 7,000 rpm for 5 min, and washed with 2 mL of phosphate-buffered saline (PBS) (1×) three times. The turbidity of the bacterial suspension was adjusted to 0.5 McFarland turbidity (1.5 × 10^8^ CFU mL^−1^; OD at 625 nm) using DMEM, measured by a spectrophotometer (CT-5000, ChromTech, Taiwan).

The cells were infected with different MOIs (10:1, 100:1, and 1,000:1) for 90 min.

The supernatant was discarded, and the cells were washed with PBS (1×). The remaining extracellular bacteria were killed with gentamicin (100 μg mL^−1^) for 1 h (Cheung and Bayles, 2007), and DMEM medium (with 1% of pen/strep) was added. The cells were then lysed with 200 μL of 0.05% SDS at 37°C for 5 min, and the suspension-containing bacteria were cultured on MHA and incubated for 24 h. The MOI was determined by counting the colonies. The MOI should be chosen such that after 24 h, no more than 20% of cell death is observed (Siegmund et al., 2021).

#### 2.11.2 Internalization assay

To determine the presence of intracellular bacteria, EA.hy926 cells were seeded in a 24-well plate (Jet Biofil, China). After 24 h of incubation, the cells were washed with PBS without calcium/magnesium (w/o Ca^2+/^Mg^2+^) and incubated with DMEM medium supplemented with 10% FBS (Siegmund et al., 2021).

The bacteria were grown in MHB at 37°C for 24 h, harvested by centrifugation at 7,000 rpm for 5 min, and washed with 2 mL of PBS (1×) three times. The turbidity of the bacterial suspension was adjusted to 0.5 McFarland standard (1.5 × 10^8^ CFU mL^−1^; OD at 625 nm) using DMEM, measured by a spectrophotometer.

EA.hy926 cells (4 × 10^4^ at 80% confluency) were infected with *S. aureus* bacteria at the predetermined MOI. This mixture was incubated at 37°C with 5% CO_2_ for 90 min. Following the incubation period, the supernatant was removed, the cells were washed with PBS (1×), and incubated with gentamycin to kill the extracellular bacteria. After 24 h, the supernatant was cultured to determine the efficiency of gentamycin in killing all extracellular bacteria.

The cell lysate was prepared as detailed above. The lysate was then serially diluted with PBS (1×) and plated on MHA. The plates were incubated at 37°C overnight, and the bacterial colonies were counted.

#### 2.11.3 Transmission electron microscopy

The TEM was used to observe the presence of intracellular bacteria in EA.hy926 cells.

The internalization of bacteria was performed as described above. Briefly, after removing the remaining bacteria, the cells were fixed in 3% glutaraldehyde (pH 7.2) for 3 h. The cells were then washed in 0.1 M PBS buffer (pH 7.2). To dehydrate the sample, increasing concentrations of ethanol were used: 25% ethanol for 10 min, 50% for 10 min, 70% for 10 min, 96% for 15 min (twice), and absolute ethanol for 15 min (twice) (Bozzola and Russell, 1999). The cells were then embedded in Spurr’s resin, thin-sectioned, stained with uranyl acetate and lead citrate, and visualized with a Zeiss EM900 transmission electron microscope (Oberkochen, Germany) at 50 kilovolts (KV).

### 2.12 Intracellular antimicrobial activity of MDP1

To assess the intracellular antimicrobial activity of MDP1, infected cells were treated with MDP1, and the numbers of intracellular bacteria were quantified by culture and confirmed by epi-fluorescent microscopy.

#### 2.12.1 Intracellular activity of MDP1

Internalization was performed as described above. The cells were washed with PBS and treated with MDP1 for 24 h, with MDP1 concentrations ranging from 0.64 to 0.04 μg mL^−1^. After treatment, the cells were lysed by adding 200 μL of 0.05% SDS, followed by incubation at 37°C for 5 min. To quantify the number of intracellular bacteria, the lysates were plated on MHA medium and incubated at 37°C overnight. Peptide-free, gentamycin-treated, infected cells served as the positive control, while non-infected cells were used as the negative control.

Although the intracellular effect of MDP1 was measured for the first time, this stage was conducted by modifying the method performed by Huo et al. (2020).

#### 2.12.2 Epi-fluorescent microscopy

To visualize the killing activity of MDP1 on intracellular *S. aureus* in EA.hy 926 cells, acridine orange and propidium iodide (PI) staining were utilized. Following the internalization of bacteria (MRSA strain) and treatment with MDP1 as described above, the cells were washed with PBS (1×) and stained with 0.01% acridine orange in PBS solution for 45 s. Subsequently, they were rinsed with PBS (1×), and stained with 0.01% PI in PBS (1×) for 45 s. Finally, the cells were observed under an epi-fluorescence microscope at 25 and 100× magnifications. Peptide-free non-infected cells were used as negative controls, respectively (Miliotis, 1991; Houalet-Jeanne et al., 2001; Thomas and Franco, 2021).

### 2.13 Statistical analysis

The results were analyzed using one-way analysis of variance (ANOVA) and *t*-test. A *p-*value ≤0.05 was considered significant. Data are presented as mean ± SD.

## 3 Results

### 3.1 Physiochemical properties

The monoisotopic molecular weight (MMW), observed molecular weight (OMW), total net charge (TNC), hydrophobicity (H), hydrophobic moment (μH), total hydrophobic ratio (THR), and GRAVY are outlined in Table 2.

**Table 2:** Physiochemical properties of MDP1.

	Peptide	L^a^	MMW (Da)^b^	OMW^c^ (Da)	TNC^d^	H^e^	μH^f^	THR^g^ (%)	GRAVY^h^
1	GIGAVLKVLTTGLPALIKRKRQQ-NH2	23	2458.55	2459.7	7	0.403	0.384	43	0.187

^a^L, peptide length (number of residues). ^b^MMW (Da), monoisotopic molecular weight (Dalton), obtained from https://peptide2.com/peptide_molecular_weight_calculator.php. ^c^OMW (Da), observed molecular weight, determined by ESI-MS. ^d^TNC, total net charge, obtained from https://aps.unmc.edu/prediction. ^e^<H>, hydrophobicity, obtained from https://heliquest.ipmc.cnrs.fr/cgi-bin/ComputParams.py. ^f^<μH>, hydrophobic moment, obtained from https://heliquest.ipmc.cnrs.fr/cgi-bin/ComputParams.py. ^g^THR (%), percentage of total hydrophobic ratio, obtained from https://aps.unmc.edu/prediction. ^h^GRAVY, grand average of hydropathicity. Obtained from https://web.expasy.org/protparam/.

### 3.2 Prediction of three-dimensional structure

**Figure 2:**
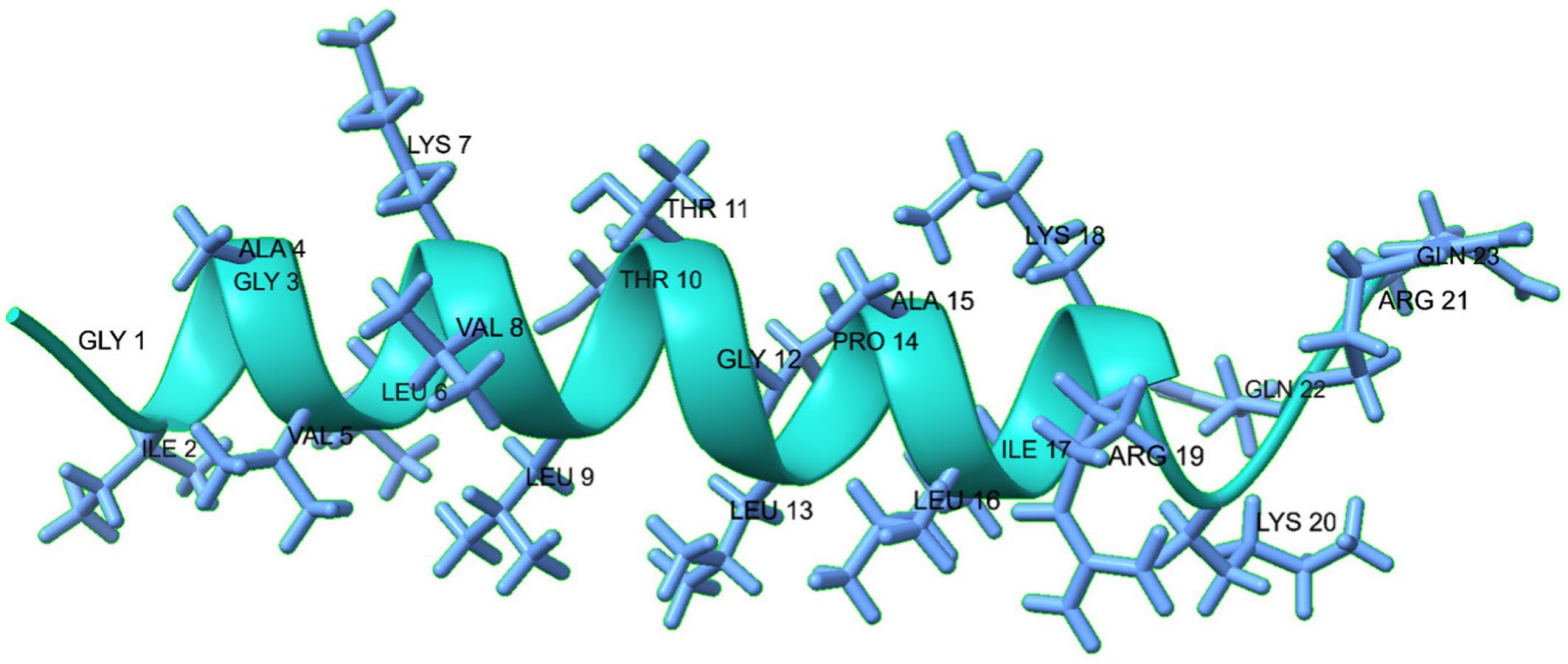
The predicted 3D structure of the MDP1 peptide, illustrates its composition of a long α- helix, and a short coil from N-terminal to C-terminal, respectively. The structure predominantly adopts an α-helical conformation, as indicated by the green color.

The MDP1 peptide is composed of a long α-helix (I2-R19: IGAVLKVLTTGLPALIKR), a short coil (K20-Q23: KRQQ), from N-terminal to C-terminal, respectively. The predicted 3D structure of MDP1 is demonstrated in Figure 2.

Protein geometry analyses showed that favored rotamers, Ramachandran favored, Rama distribution *Z*-score, MolProbity score, and Cβ deviation were, respectively, 94.44, 95.24%, −1.5 ± 1.57, 1.40, and 0 (Supplementary Figure S3). The data indicates the accuracy of the structure prediction of MDP1.

### 3.3 Molecular dynamics simulation

#### 3.3.1 Mechanism of interaction

**Figure 3:**
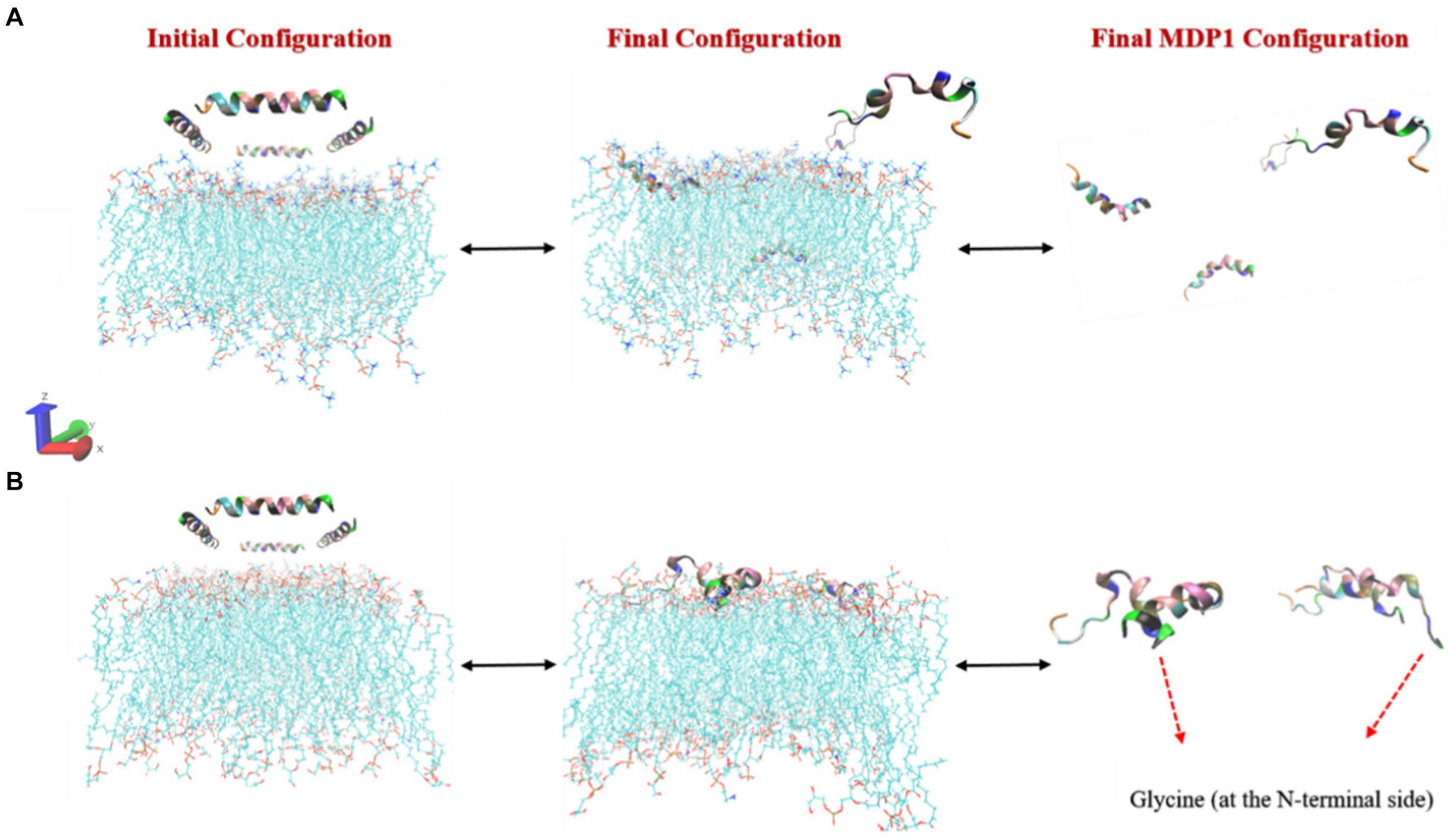
Initial and final configurations of MDP1 toward **(A)** POPC and **(B)** POPG/POPE membranes.

The interaction of MDP1 with the POPC membrane indicates that out of the four MDP1 peptides placed in the system, two fully interacted with the membrane while the other two migrated toward the aqueous phase without any interaction, as shown in Figure 3. Among the interacting peptides, one penetrated deeply into the membrane almost reaching the mid-region, mimicking the behavior of transmembrane passage, while the other exhibited significant penetration. The final state of the peptides showed that the two penetrating peptides largely retained their initial helical structure and penetrated the membrane from the glycine (at the N-terminal) end. The other two MDP1 peptides, which did not show effective interaction with the POPC membrane, exhibited noticeable structural changes compared to their initial state. The interaction of MDP1 with the POPG/POPE membrane was significantly more pronounced with all four MDP1 peptides interacting with the membrane. Two MDP1 peptides specifically and effectively interacted with the membrane from the glycine end of the peptides, while the sequence at the other end of the MDP1 appeared to deviate somewhat from its initial helical structure. The peptide partially unfolded from the C-terminal end, providing the necessary driving force for membrane penetration. The interaction of the other two MDP1 peptides also occurred from the same end, accompanied by a slight unfolding of the peptide’s initial helical structure in this region.

#### 3.3.2 Interaction energies

**Figure 4:**
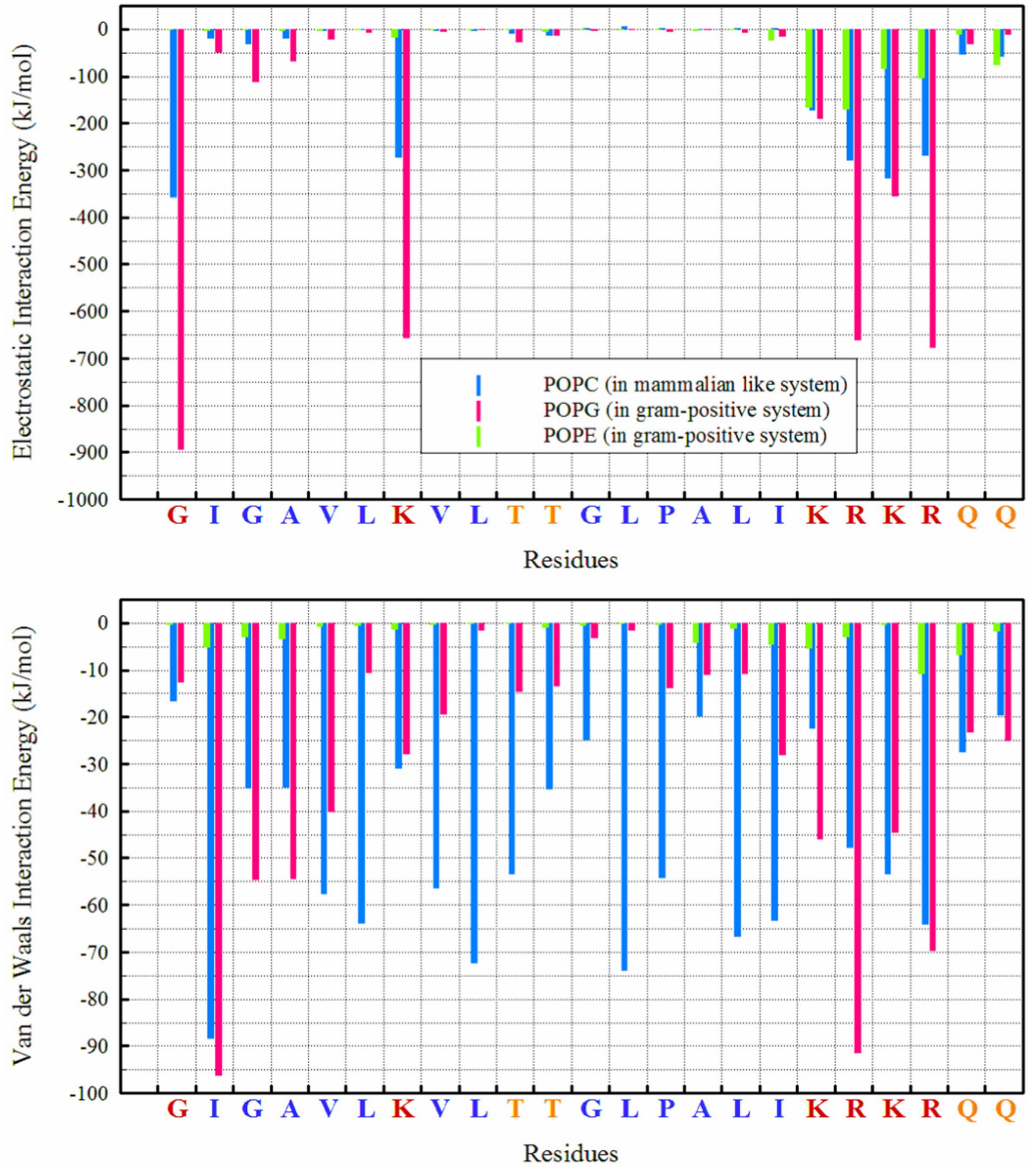
Interaction energies between different lipids and residues in POPC and POPG/POPE membranes.

From a thermodynamic perspective, examining the interaction energy between system components can provide a clear picture of the thermodynamics of interactions a system (Boroushaki et al., 2023; Ganjali Koli and Fogolari, 2023; Gholami et al., 2024) and the preferred orientation of peptides relative to membranes. Figure 4 clearly shows that the dominant energy in the interaction of MDP1 with the POPC membrane is electrostatic (−1850 kJ mol^−1^), accounting for more than 63% of the total interaction energy (−2930.34 kJ mol^−1^). Charged residues such as lysine (K), arginine (R), and glycine (G at the N-terminal) play a major role in this context. Additionally, van der Waals (vdW) interactions also contribute significantly (−1080.33 kJ mol^−1^), comprising about 37% of the total interaction energy. Hydrophobic residues (G, A, V, L, I, P) exhibit strong vdW interactions, indicating better compatibility of these residues with the POPC membrane.

However, the electrostatic interaction energy of MDP1 with the POPG/POPE membrane is significantly higher than with POPC. Electrostatic interactions (−4444.8 kJ mol^−1^) account for more than 85% of the total interaction energy (−5208.8 kJ mol^−1^). POPG plays a major role due to its negatively charged head group (−3792.05 kJ mol^−1^), while POPE has a smaller contribution due to its zwitterionic head group (−652.8 kJ mol^−1^). The vdW interactions contribute less (less than 15%) to the total interaction energy (−763.97 kJ mol^−1^) compared to the POPC membrane.

#### 3.3.3 Hydrogen bond

Hydrogen bonds play a crucial role in the interactions between peptides and proteins, as well as with membrane lipids (Hesamzadeh et al., 2024). They stabilize binding, facilitate the proper orientation and insertion of peptides into the lipid bilayer, and thereby influence the structure and function of both the peptide and the membrane, particularly for interactions with POPG, as seen in Figure 1. The highest number of hydrogen bonds is observed between ‘R’ residues, followed by ‘G’ (in the N-terminal region) and ‘K’ with POPG. In the POPC membrane, a wider range of residues are capable of forming hydrogen bonds; in addition to the KRKR sequence, the first two residues on the N-terminal side (G and I) and the K residue contribute significantly to hydrogen bond formation. The total number of hydrogen bonds observed for POPG, POPE, and POPC were 42.88, 7.45, and 19.69, respectively.

#### 3.3.4 Secondary structure

As indicated in Table 3 and Supplementary Figure S4, the secondary structures of MDP1 molecules in various simulation systems were determined using DSSP (Kabsch and Sander, 1983; Touw et al., 2014). The secondary structure of MDP1 molecules provides valuable insights into their folding preferences across different lipid environments. In POPC membranes, the peptides predominantly adopt α-helical structures, with α-helix content ranging significantly from 27 to 78%, indicating substantial stabilization of helical conformations in this lipid environment. MDP1_1 and MDP1_2 exhibit the highest α-helix content at 73 and 78%, respectively, due to their strong interaction with the membrane. In contrast, MDP1_3 and MDP1_4 display more diverse distributions, with α-helix contents of 50 and 27%, respectively, suggesting variations in helical propensity among the peptides when go through the bulk of water. In the POPG/POPE system, α-helix content tends to be lower and less variable across peptides, averaging between 49 and 57%, that could be attributed to interactions with the membrane components. This indicates relatively stable helical structures in mixed bilayers influenced by negatively charged POPG and different packing properties of POPE. MDP1_1 and MDP1_2 show α-helix contents of 55 and 56%, respectively, in POPG/POPE, while MDP1_3 and MDP1_4 exhibit α-helix contents of 49 and 57%, respectively. This suggests a slightly lower but consistent preference for helical structures compared to POPC. Additionally, different peptides and lipid systems display varying percentages of secondary structure elements such as coils, bends, and turns. In the POPC system, coil content ranges from 13 to 45%, while in POPG/POPE, it varies from 21 to 27%. Bend percentages range from 2 to 17% in POPC and from 3 to 11% in POPG/POPE. Turn structures remain relatively stable in both systems, with percentages ranging from 4 to 7% in POPC and from 8 to 13% in POPG/POPE. The presence of 3-helices and 5-helices, although limited, also fluctuates, underscoring the lipid-dependent variations in peptide secondary structure.

**Table 3:** The averaged population of the secondary structure content from DSSP analysis.

	Number of Peptide	Coil (%)	Bend (%)	Turn (%)	α-Helix (%)	3-Helix (%)	5-Helix (%)
In POPC system	MDP1_1	15	3	5	73	2	1
	MDP1_2	13	2	4	78	0	2
	MDP1_3	34	9	5	50	1	0
	MDP1_4	45	17	7	27	4	0
In POPG/POPE system	MDP1_1	25	11	8	55	0	2
	MDP1_2	21	3	9	56	4	6
	MDP1_3	27	9	13	49	1	0
	MDP1_4	22	6	7	57	1	7

### 3.4 Antimicrobial activity of peptide

MDP1 inhibited the growth of all *S. aureus* isolates or eradicated them as shown in Table 4.

**Table 4:** MIC and MBC values for the examined *S. aureus* isolates.

	MRSA1	VRSA2	ATCC
MIC	8 μg mL^−1^	8 μg mL^−1^	0.5 μg mL^−1^
MBC	8 μg mL^−1^	16 μg mL^−1^	0.5 μg mL^−1^

### 3.5 Circular dichroism spectroscopy

**Figure 5:**
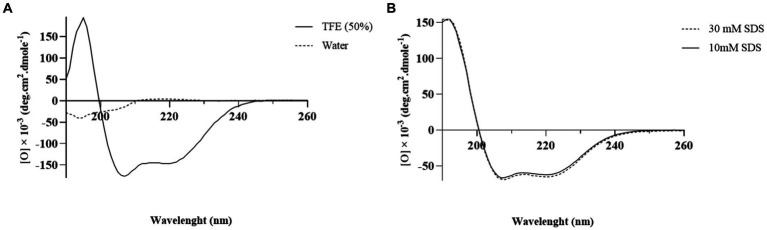
The CD spectrum analysis of MDP1. **(A)** TFE 50% and water **(B)** SDS 10 and 30 mM. Analysis of the results showed that the helical content of the peptide was 52.5, 87, and 91% in TFE 50%, SDS 10, and 30 mM, respectively.

The CD spectrum of MDP1 showed a single positive band at 190 nm and double minimum bands at 208 and 218 nm, which is characteristic of an α-helical structure (Figure 5). The helical content of the peptide in the SDS concentrations of 10 and 30 mM were, respectively, 87 and 91%. The results of 2D determination for MDP1 in TFE 50%, obtained from Akbari et al. (2018) study, was represented.

### 3.6 Selection of the most pathogenic *S. aureus*

**Figure 6:**
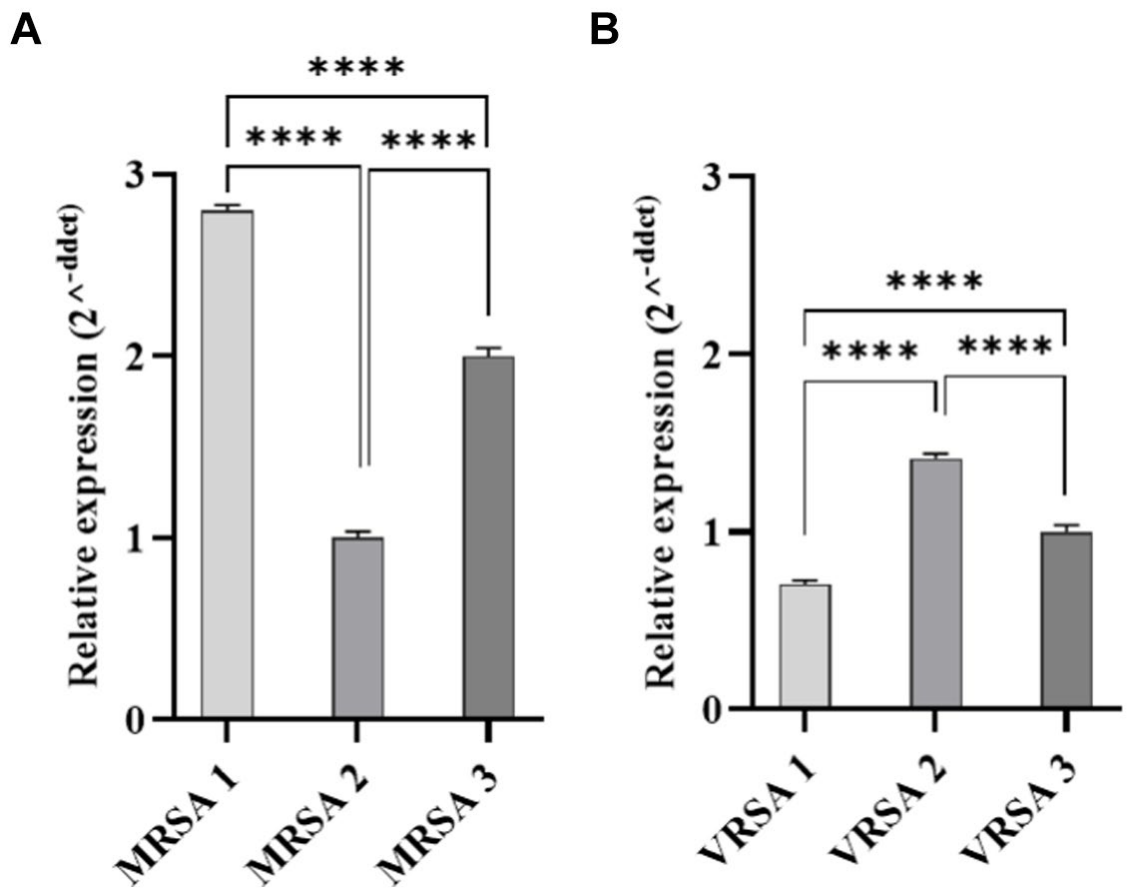
Quantitative Real-time PCR analysis of *fnbpA* expression in clinical isolates of *S. aureus*. **(A)** Represents MRSA isolates, while **(B)** represents VRSA isolates. Statistical analysis revealed a significant difference in *fnbpA* expression levels between MRSA and VRSA isolates. ****p < 0.0001.

Gene expression of *fnbpA* was determined in selected MRSA and VRSA clinical isolates. Real-time PCR revealed statistical difference in the amount of *fnbpA* expressed by MRSA and VRSA isolates (*p-*value <0.0001; Figure 6). MRSA1 and VRSA2 had the highest level of *fnbpA* expression and were selected for further assays.

### 3.7 Evaluation of MDP1 toxicity on EA.hy 926 cell

**Figure 7:**
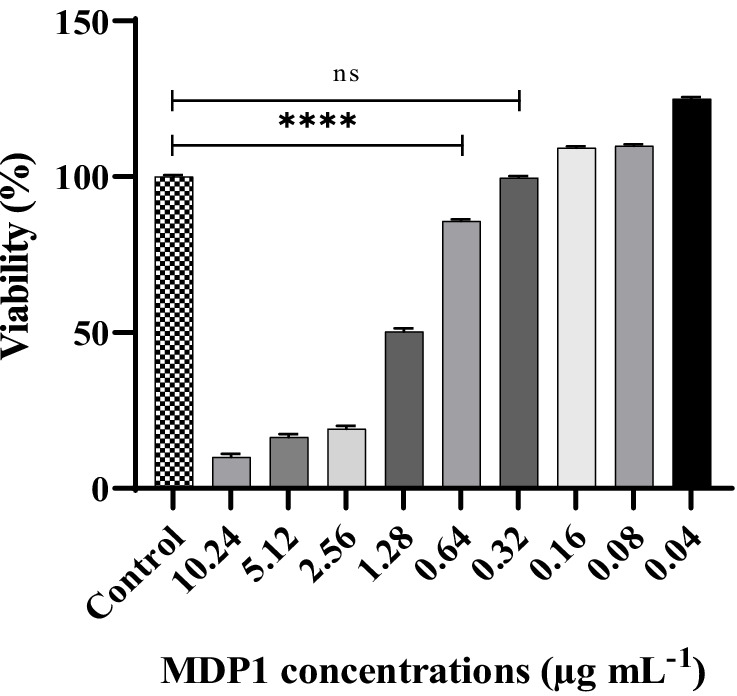
Cytotoxicity assessment of MDP1 against EA.hy 926 cells across varying concentrations (ranging from 10.24 to 0.04 μg mL^−1^). Notably, cells treated with MDP1 at the concentration of 0.32 μg mL^−1^ exhibited 100% viability, indicating no cytotoxic effects. Untreated cells were used as the negative control (NC). According to 10,993–12 standards (Standardization, 2021), compounds are non-cytotoxic if their viabilities are ≥70% of the control group. ****p < 0.0001.

EA.hy 926 cells were exposed to varying concentrations of MDP1 (ranging from 10.24 to 0.04 μg mL^−1^). As depicted in Figure 7, the cells treated with MDP1 at a concentration of 0.32 μg mL^−1^ exhibited 100% viability.

### 3.8 Localization of MDP1 on *S. aureus*

**Figure 8:**
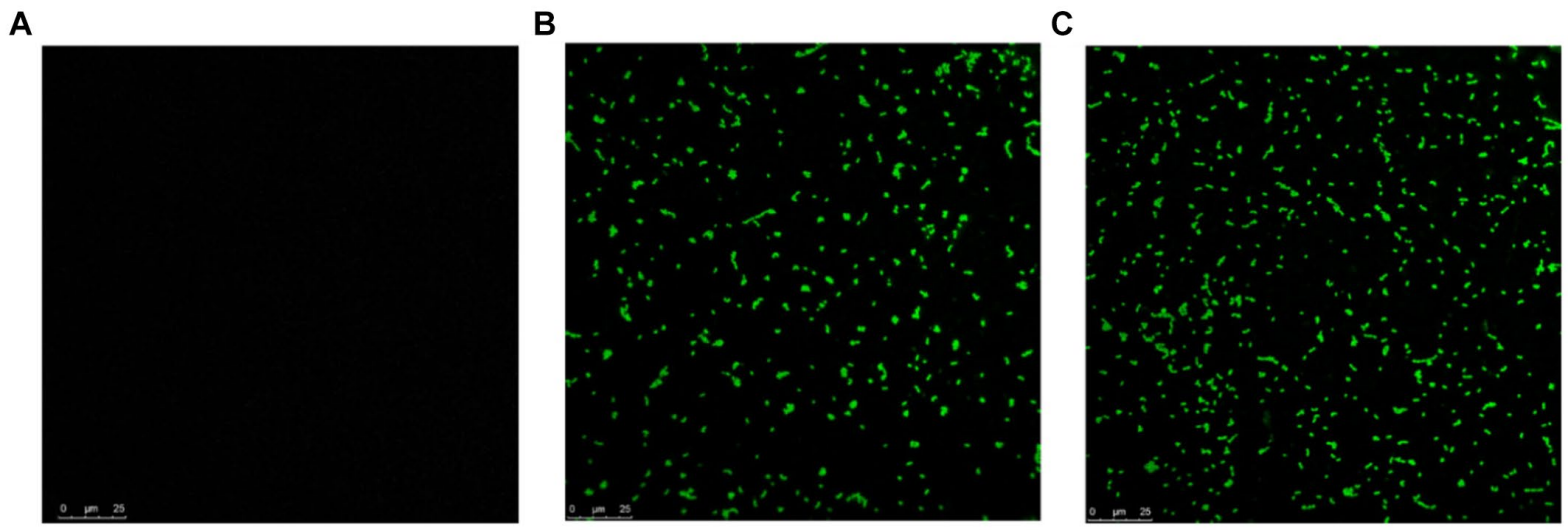
CLSM observation for localization of FITC-labeled MDP1 on *S. aureus*. **(A)** Untreated *S. aureus.* **(B)** MRSA and **(C)** VRSA were co-incubated with 0.32 μg mL^−1^ FITC-labeled MDP1 for 1 h, and observed with CLSM.

To determine if MDP1 can bind to *S. aureus*, the antibacterial mechanism of MDP1 against *S. aureus* was further investigated using CLSM. As shown in Figure 8, fluorescence signals were detected on the surface of *S. aureus* treated with FITC-labeled MDP1, indicating that MDP1 can bind to bacterial cells.

### 3.9 Induction of cell disruption by MDP1

**Figure 9:**
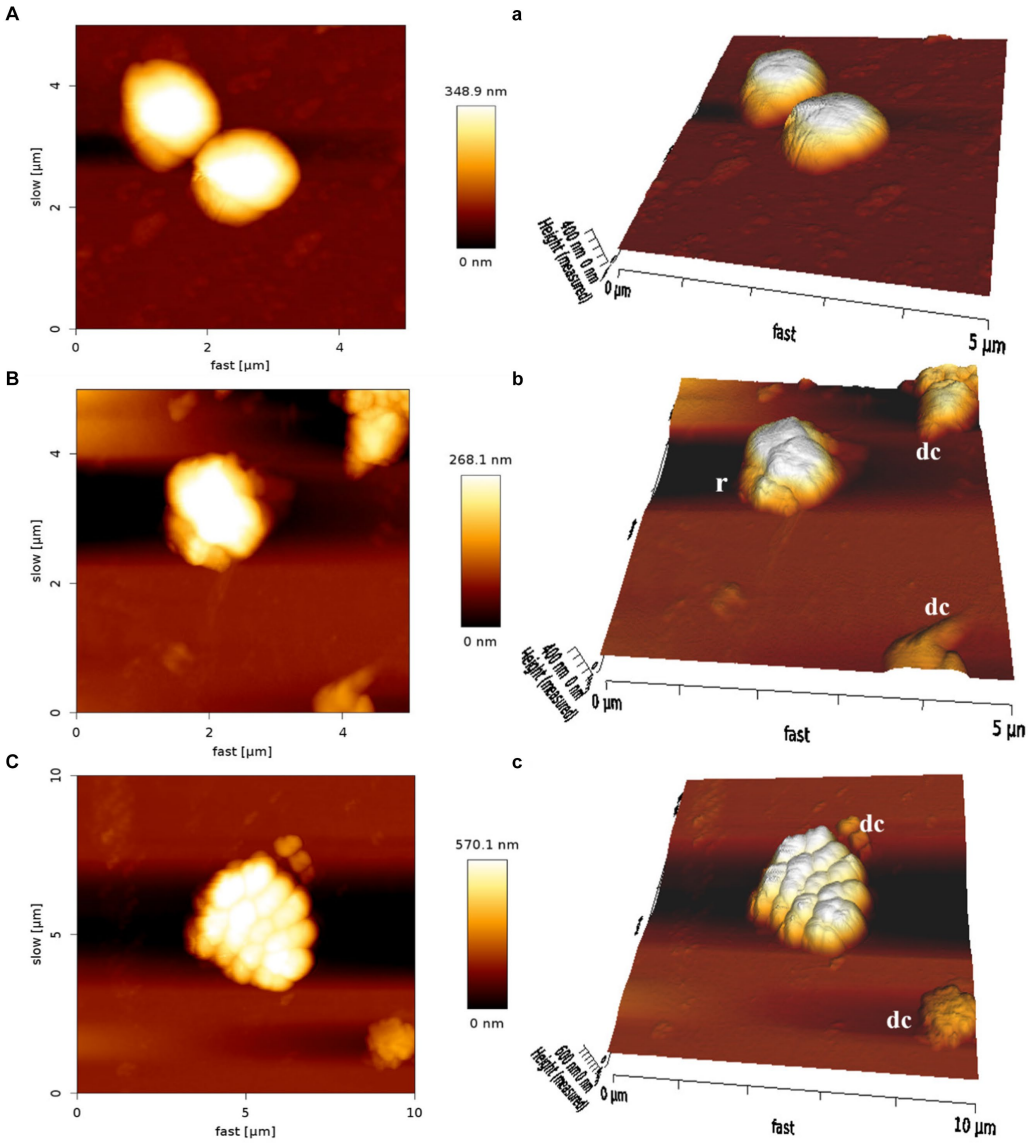
Atomic force microscopy of *S. aureus*. Untreated *S. aureus* (**A**: control) or in the presence of peptide (**B**: VRSA) (**C**: MRSA). An analysis of non-contact mode images was presented as topography **(A–C)** and 3D images **(a–c)**. r, roughness; dc, dead cells.

The AFM was employed to visualize the potential damage induced by MDP1 on the *S. aureus* membrane. At the concentration of 0.32 μg mL^−1^, MDP1 caused slight morphological changes, evident as increased surface roughness in *S. aureus* and even death, compared to the untreated control (Figure 9).

### 3.10 Evaluation of *S. aureus* internalization

#### 3.10.1 Determination of MOI

The determination of the number of *S. aureus* used for cell infection was investigated by infecting EA.hy 926 cells at different MOIs of 10, 100, and 1,000. Our results indicated the optimum infection was achieved in the cells when MOI = 100, which is approximately 5 × 10^3^ CFU mL^−1^.

#### 3.10.2 The localization of intracellular *S. aureus* by TEM

**Figure 10:**
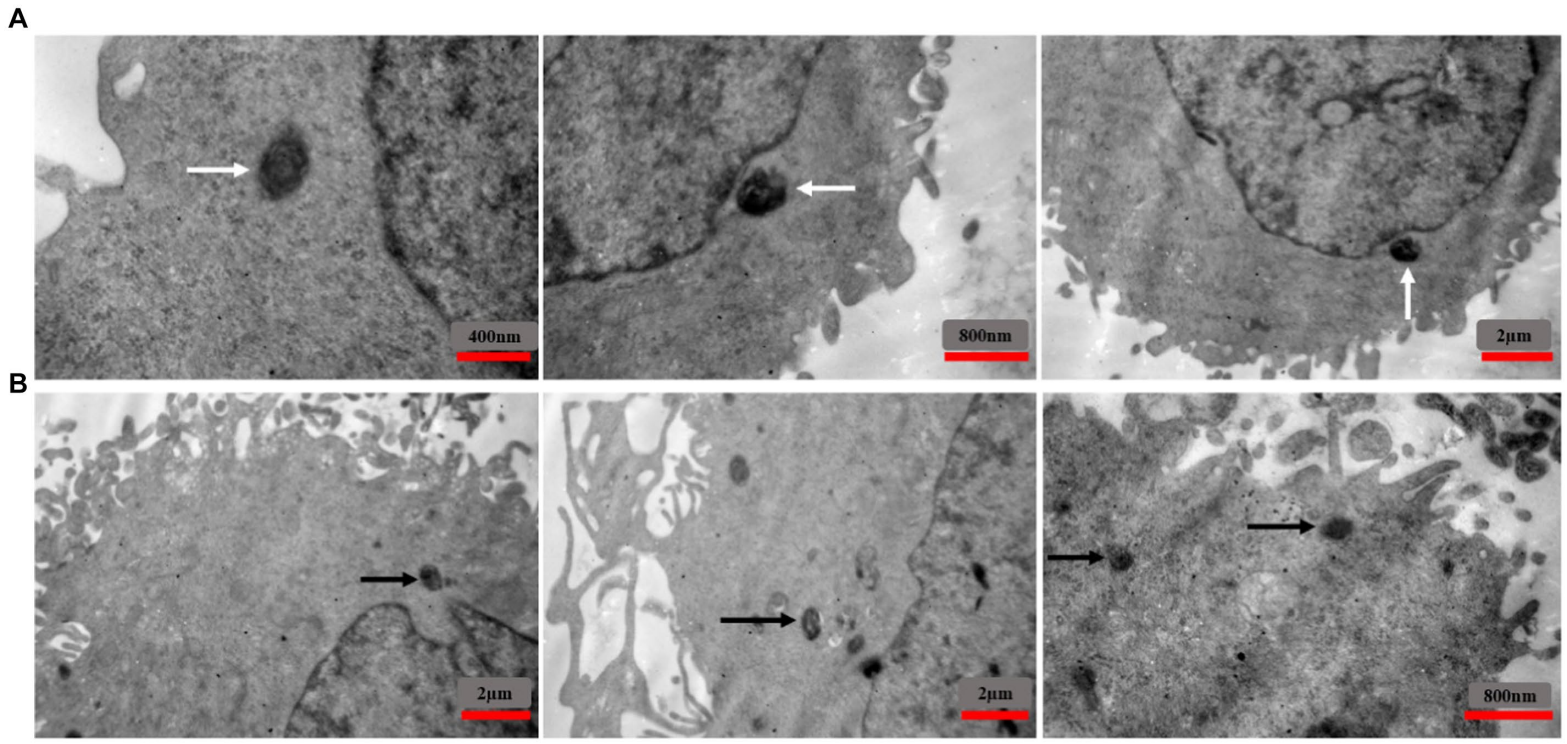
TEM images showing the intracellular localization of MRSA1 and VRSA2 strains within EA.hy 926 cells. The results indicate that both MRSA and VRSA strains can enter cells without causing host cell damage, with intracellular bacteria primarily located in the cytoplasm. White arrows **(A)** indicate MRSA strains, while black arrows **(B)** indicate VRSA strains.

To investigate the localization of intracellular *S. aureus*, TEM was used to analyze EA.hy 926 cells infected with MRSA1 and VRSA 2 (Figure 10). The results showed that the pathogen could enter the cells without damaging the host cells and that the intracellular bacteria were located in the cytoplasm.

### 3.11 Intracellular antimicrobial activity of MDP1

#### 3.11.1 Intracellular activity of MDP1

*Staphylococcus aureus* can invade and persist within mammalian cells, posing significant challenges in treating intracellular infections. The study focused on the intracellular bactericidal effects of MDP1 using *S. aureus* as a model. The reduction of intracellular bacterial load after MDP1 treatment is outlined in Table 5.

**Table 5:** Intracellular bacterial load after MDP1 treatment.

MDP1 concentration	0.32 μg mL^−1^		
Number of bacteria	Test (CFU mL^−1^)	PC^*^ (CFU mL^−1^)	NC^#^ (CFU mL^−1^)
Bacteria			
ATCC	230 ± 8	5,000 ± 37	0
VRSA2	3,005 ± 17	5,136 ± 29	0
MRSA1	715 ± 12	5,218 ± 31	0

*PC, positive control (peptide-free, gentamycin-treated, infected cells), ^#^NC, negative control (peptide-free non-infected cells).

**Figure 11:**
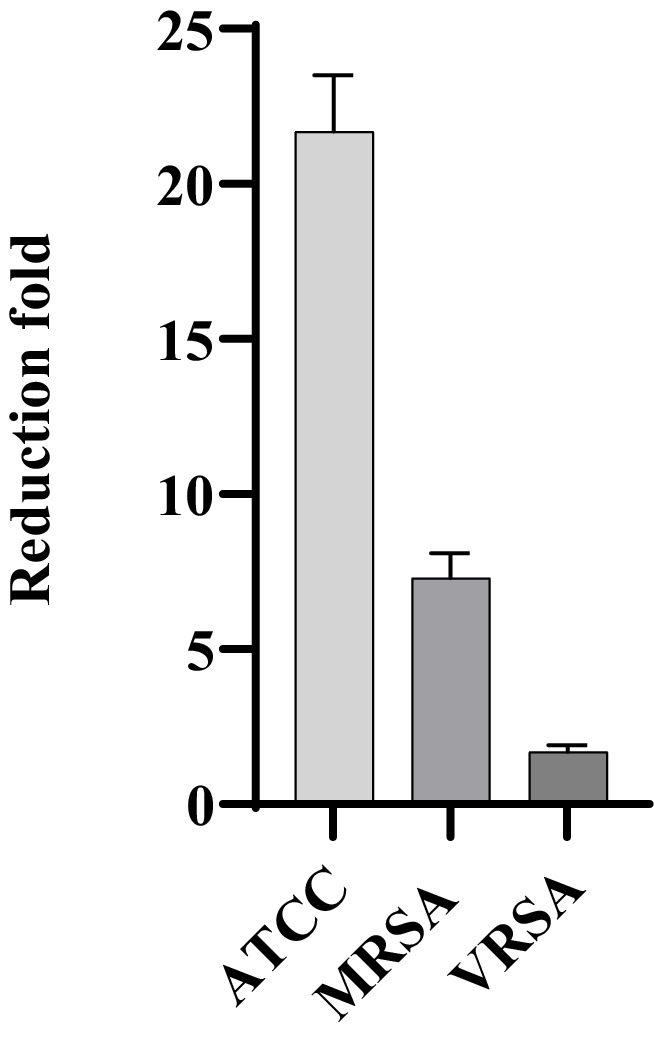
Reduction in the number of bacteria. MDP1, at the concentration of 0.32 μg mL^−1^, demonstrated a fold reduction of 21.7 ± 1.8, 1.7 ± 0.2, and 7.3 ± 0.8 in intracellular bacterial load for ATCC, VRSA, and MRSA, respectively. The fold reduction values represent the relative decrease in bacterial load.

MDP1, at the concentration of 0.32 μg mL^−1^, demonstrated a fold reduction of 21.7 ± 1.8, 1.7 ± 0.2, and 7.3 ± 0.8 in intracellular bacterial load for ATCC, VRSA2, and MRSA1, respectively, resulting in a substantial decrease in intracellular *S. aureus* cells compared to the positive control conditions (Figure 11). This significant fold reduction highlights the efficacy of MDP1 in eliminating intracellular bacteria, emphasizing its potential as an effective antimicrobial agent in combating intracellular infections. Comparison of the results between the test and control groups showed significant differences (*p*-value ≤0.001).

#### 3.11.2 Epi-fluorescent microscopy

**Figure 12:**
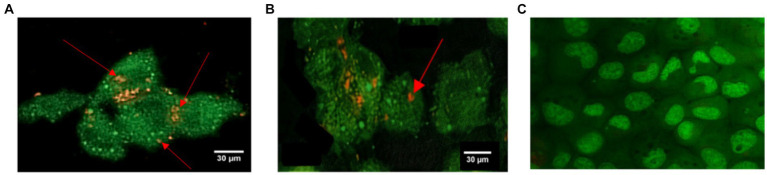
Assessment of intracellular *S. aureus* viability within EA.hy 926 cells following exposure to MDP1 at 0.32 μg mL^−1^ using acridine orange and PI staining. **(A,B)** Fluorescence microscopy images revealing all invading bacteria absorbing acridine orange and exhibiting green fluorescence, indicating viable intracellular organisms, with nonviable bacteria exhibiting orange staining due to the heightened interaction with PI. **(A)** MRSA, **(B)** VRSA, and **(C)** non-infected cells as negative controls.

*Staphylococcus aureus* was assessed in infected EA.hy 926 cells after 24 h treatment with MDP1 at 0.32 μg mL^−1^. All invading bacteria absorbed acridine orange and exhibited green fluorescence. The use of the acridine orange staining method revealed viable intracellular organisms fluorescing in green (Figure 12). The color shift is associated with the heightened interaction of acridine orange with the phosphate-sugar backbone of denatured DNA in non-viable cells (West, 2006). In the case of non-viable intracellular bacteria, the staining would have appeared orange with PI. Fluorescence microscopy studies support the intracellular bactericidal effect of MDP1 on intracellular *S. aureus* after treatment with MDP1, although it cannot be effective in the eradication of bacteria in cells.

## 4 Discussion

Prediction of physiochemical properties of MDP1 showed TNC of 7 and hydrophobic ratio of 43% which indicate the proper features as an AMP to be interacted with bacterial membranes. The positively charged residues including lysine (K) and arginine (R) are known to interact with the negatively charged components of bacterial membranes. It’s possible that the higher positive charge of certain residues, particularly arginine, contributes to their stronger interaction with the negatively charged bacterial membranes, resulting in greater antimicrobial activity. On the other hand, the bacterial cell membrane has a more negative charge compared to eukaryotic cell membranes. This difference in charge is primarily due to the composition of the cell membranes. Bacterial cell membranes contain teichoic acids (in Gram-positive bacteria) in their outer layers, which contribute to the negative charge. These molecules contain phosphate groups and other negatively charged components that confer an overall negative charge to the bacterial cell membrane. In contrast, eukaryotic cell membranes are composed mainly of phospholipids, which do not confer as much negative charge as the components found in bacterial membranes. Additionally, eukaryotic cells may have surface glycoproteins and glycolipids, but these do not typically contribute as significantly to the overall negative charge of the cell membrane. Therefore, the higher negative charge of bacterial membranes compared to eukaryotic cell membranes is one of the factors that make AMPs, which often carry positive charges, more selective in targeting bacteria while having minimal effects on eukaryotic cells.

The results obtained from three-dimensional structural prediction showed that MDP1 has α-helical conformation which is roughly similar to the results of CD spectroscopy in membrane mimetic environments like SDS micelles.

The results of molecular dynamics simulations revealed that the interaction mechanism of MDP1 with POPC and POPG/POPE membranes highlights the differential behavior of the peptide in different lipid environments. The deeper penetration of MDP1 into the POPC membrane suggests a strong affinity driven by the peptide’s structural properties. In contrast, the complete interaction of MDP1 with the POPG/POPE membrane indicates that electrostatic interactions are the primary driving force for this binding, especially with the negatively charged POPG.

The interaction energy analysis provides a thermodynamic perspective on the binding preferences of MDP1 with different membranes. The significant electrostatic interaction energy with POPG/POPE compared to POPC underscores the importance of charge interactions in peptide-membrane binding. The lesser contribution of vdW interactions in the POPG/POPE system compared to POPC indicates that electrostatic interactions are more crucial for binding to negatively charged membranes. It appears that the presence of “CH₃” groups in the head group of POPC enhances vdW interactions of MDP1 residues when interacting with POPC. In the POPC membrane, residues like glycine (G), alanine (A), valine (V), leucine (L), threonine (T), and isoleucine (I) show relatively small or negligible electrostatic interactions, consistent with their neutral or non-polar nature.

In POPG/POPE membrane, the electrostatic interactions of charged residues lysine (K), arginine (R), and glycine (G at the N-terminal) of MDP1 with POPG are the driving force for binding to the membranes of Gram-positive bacteria.

Hydrogen bonding patterns further elucidate the binding mechanisms of MDP1 with different membranes. The higher number of hydrogen bonds with POPG correlates with the stronger electrostatic interactions observed, reinforcing the role of hydrogen bonds in stabilizing peptide-membrane interactions. The ability of a broader range of residues to form hydrogen bonds with POPC indicates a more diverse interaction profile compared to POPG/POPE.

The secondary structure analysis reveals the influence of lipid composition on the folding and stability of MDP1. The higher α-helix content in POPC suggests a stabilizing effect of this lipid environment on helical structures. Conversely, the mixed POPG/POPE membrane induces more variability in secondary structure, likely due to the different packing properties and electrostatic environment provided by these lipids.

MDP1 showed no toxicity at the concentration of 0.32 μg mL^−1^ when tested by MTT assay. The result of our cytotoxicity assay diverged from those reported in the previous study (Akbari et al., 2022). The variations in the results of the cytotoxicity assay may be, in part, attributable to the use of different cell types. Cells exhibit distinct sensitivities to external agents, including peptides, due to variations in their surface receptors, intracellular pathways, different membrane compositions, and physiological properties. The choice of cell type can significantly influence the observed cytotoxicity of the peptides. MDP-1 exhibited significant antibacterial efficacy against the examined extracellular *S. aureus*, including ATCC and MDR strains (Akbari et al., 2018). To identify the intracellular activity of the peptide against *S. aureus*, a non-toxic concentration of the peptide was applied to the infected cells. Our findings demonstrated that MDP1 reduced the number of intracellular bacteria.

Following molecular dynamics simulation, experimental testimonies including confocal microscopy and AFM proved the MDP1-gram positive bacterial membrane interaction. The results of AFM regarding the alteration in bacteria morphology by MDP1 are in accordance with SEM results in Akbari et al. (2018) study. Potent calcein release from MRSA and VRSA strains by MDP1 in a non-toxic dose (0.32 μg mL-1) in Akbari et al. (2018) study is also in line with confocal microscopy observation in our study.

Internalization of *S. aureus* bacteria was confirmed by culture and TEM and their elimination by MDP1 was then approved by culture and Epi-Fluorescent microscopy. MDP1 succeeded in eliminating the intracellular bacteria partially.

To the best of our knowledge, no study has investigated the AMP’s effect on intracellular *S. aureus* in endothelial cell lines. However, there are studies that have investigated the effects of AMPs on other cells and bacteria. Huo et al. (2020) study highlights the potent antibacterial activity of TAT-KR-12, a cell-penetrating peptide derived from the fusion of the trans-activating transcription (TAT) peptide and residues 18–29 of human cathelicidin LL-37 (KR-12), against clinical strains of *S. aureus* using RAW264.7 cells. TAT-KR-12 was also effective in eliminating the intracellular *S. aureus* cells *in vitro*, indicating high anti-intracellular characteristics (Huo et al., 2020). Another study demonstrated that linking the N2 peptide with the cell-penetrating peptides bLFcin6 or TAT11 increased its effectiveness in killing internalized *Salmonella typhimurium* (Li et al., 2018). Although a direct comparison of these studies with ours may not be feasible due to the different behavior of cells and bacteria, what brings these studies closer to ours is the peptide’s structure, which has been able to enter the cell without causing harm to eukaryotic cells and exert its antimicrobial effect.

Nepal et al. (2018) discovered three cationic amphiphilic polyproline helices with strong cell penetration in macrophages and potent antibacterial effects against intracellular Gram-positive and Gram-negative bacteria. In another study, a synthetic peptide effectively targets the cell membrane of intracellular bacteria due to its hydrophobic tryptophan-rich motif and hydrophilic lysine-rich domain (Park et al., 2009).

Future clinical applications of MDP1 for treating intracellular infections, particularly those caused by *S. aureus*, contact with some significant challenges, i.e., cost-ineffectiveness, off-target binding, and possible toxicity. Current pharmacokinetic data and clinical toxicity profiles indicate that a large amount of peptide would need to be administered intravenously to effectively eliminate the intracellular bacteria. This presents a dual challenge: cost-ineffectiveness and a risk of potential toxicity to the human body.

None of the conducted studies were successful in complete eradication of intracellular bacteria. In this regard, Huo et al. (2020) suggested using CPP to increase the intracellular bioavailability of the desired AMP but it could not guarantee the complete eradication of bacteria. Thus, this issue is still to be resolved. Concerning this problem, we suggest targeting specific biomarkers to enhance the delivery of AMP to the cytoplasm by receptor-mediated endocytosis (RME). Practically, a unique integral protein in the membrane of infected cells, as a cell-specific biomarker, would be targeted by an antibody-AMP conjugate to enhance the delivery of desired AMP to the cytoplasm by RME. This targeted delivery method holds promise for enhancing the effectiveness of the peptide while simultaneously simplifying the process of intravenous administration. By specifically targeting receptors on endothelial cells, the need for large quantities of peptides for effective treatment may be reduced, potentially addressing the cost-effectiveness concern. Additionally, this targeted approach could minimize the risk of systemic toxicity by ensuring that the peptide is delivered primarily to the intended site of action.

## 5 Conclusion

The intracellular antibacterial activity of MDP1 within endothelial cells underscores its potential as a therapeutic agent against intracellular pathogens. The membrane binding affinity of MDP1 highlights its ability to effectively interact with the cellular membranes of endothelial cells and its intracellular action against *S. aureus*.

A molecular dynamics study elucidates the selective interaction of MDP1 peptides with bacterial-like membranes, highlighting the preferential engagement with POPG/POPE over POPC membranes. Interaction energies were significantly higher for POPG/POPE (−5208.8 kJ mol^−1^) compared to POPC (−2930.34 kJ mol^−1^), with electrostatic interactions dominating in both cases. Electrostatic forces emerge as the primary driver of this selectivity, particularly due to interactions between positively charged MDP1 residues and negatively charged POPG lipids. Hydrogen bond analysis corroborated these findings, showing a higher number of bonds with POPG, which further supports MDP1’s potential specificity for bacterial targets.

Secondary structure analysis revealed that lipid composition influences MDP1 conformation, with POPC promoting higher α-helical content. The adaptation of the MDP1 secondary structure to different lipid environments suggests a flexible mechanism of action that could be crucial for antimicrobial activity.

This finding shed light on the mechanism of action of MDP1 although further studies should be performed to increase the intracellular activity of MDP1.

## Data Availability

The original contributions presented in the study are included in the article/Supplementary material, further inquiries can be directed to the corresponding authors.

## Footnotes

^1^ http://web.expasy.org/protparam

^2^ https://aps.unmc.edu/prediction

^3^ https://zhanggroup.org/I-TASSER

^4^ http://molprobity.biochem.duke.edu/

## Glossary

MDP1: Melittin derived peptide 1
VRSA: Vancomycin resistant *S. aureus*
MRSA: Methicillin-resistant *S. aureus*
TEM: Transmission electron microscopy
FnBPA: Fibronectin-binding proteins A
FnBPB: Fibronectin-binding proteins B
AMPs: Antimicrobial peptides
CPPs: Cell-penetrating peptides
SDS: Sodium dodecyl sulfate
Pen-Strep: penicillin–streptomycin
MHB: Mueller-Hinton Broth
MHA: Mueller−Hinton Agar
DMEM: Dulbecco’s Modified Eagle Medium
FBS: Fetal Bovine Serum
BCA: Bicinchoninic acid assay
RMSD: Root mean square deviation
ADT: AutoDock Tools
RP-HPLC: Reverse-phase high-performance liquid chromatography
MIC: Minimum Inhibitory Concentration
MBC: Minimum Bactericidal Concentration
OD: optimal density
CLSI: Clinical and Laboratory Standards Institute
MOI: multiplicity of infection
PBS: Phosphate buffer saline
PI: propidium iodide
MMW: Monoisotopic molecular weight
OMW: Observed molecular weight
TNC: Total net charge
H: Hydrophobicity
μH: Hydrophobic moment
THR: Total hydrophobic ratio
GRAVY: Grand average of hydropathicity
ICAM-1: Intercellular Adhesion Molecule 1
CD: Circular dichroism
AFM: Atomic force microscopy
