# Supplementary material for: Intracellular bactericidal activity and action mechanism of MDP1 antimicrobial peptide against VRSA and MRSA in human endothelial cells
Source: Front Microbiol. 2024 Aug 26;15:1416995. doi: 10.3389/fmicb.2024.1416995 (PMC11381295; doi:10.3389/fmicb.2024.1416995)
Supplement: Supplementary file 1 [file Data_Sheet_1.PDF]

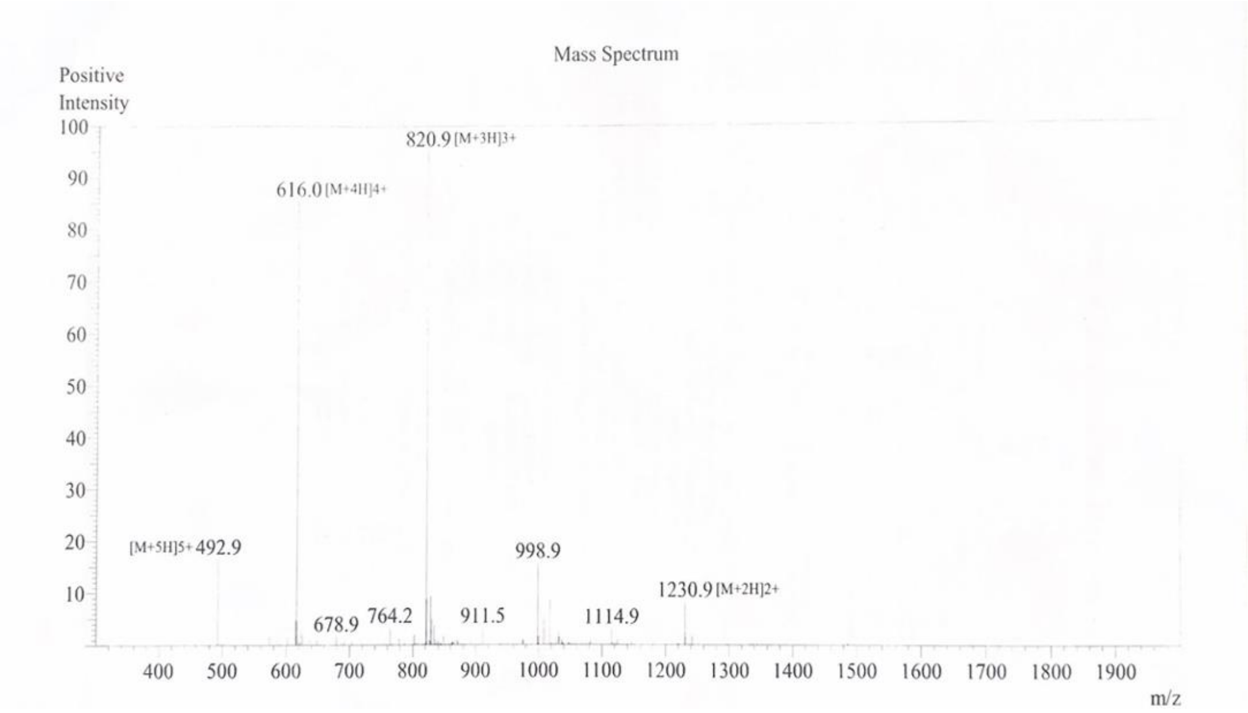

Figure S1. The mass spectra report of MDP1.

Pump A : 0.065% trifluoroacetic in 100% water (v/v)
Pump B : 0.05% trifluoroacetic in 100% acetonitrile (v/v)
Total Flow:1 ml/min
Wavelength:220 nm
<<LC Time Program>>

Time	Module	Command	Value
0.01	Pumps	B.Conc	5
25.00	Pumps	B.Conc	65
25.01	Pumps	B.Conc	95
27.00	Pumps	B.Conc	95
27.01	Pumps	B.Conc	5
35.00	Pumps	B.Conc	5
35.01	Controller	Stop	

<<Column Performance>>
<Detector A>
Column :Inertsil ODS-3 4.6 x 250 mm
Equipment: ZJ20010139

<Chromatogram>

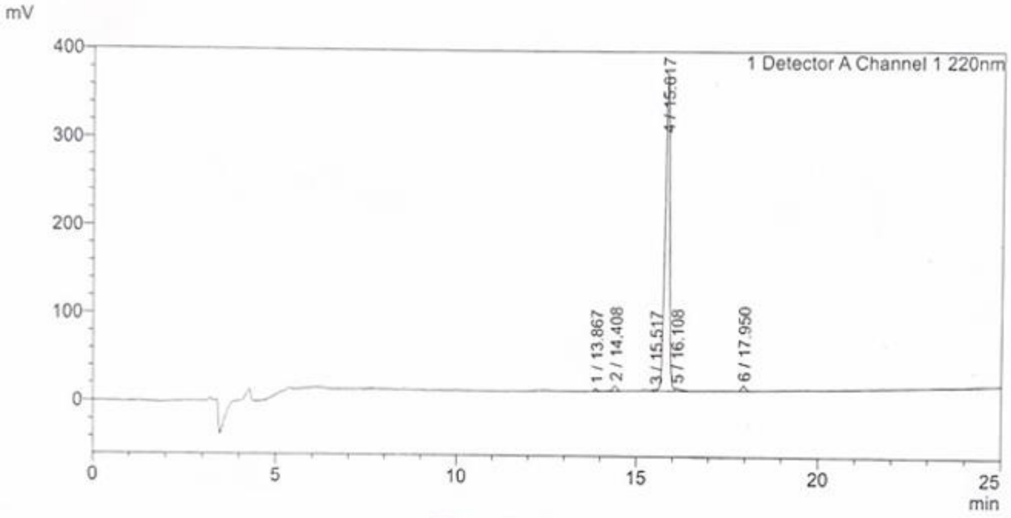

<Peak Table>

Detector A Channel 1 220nm

Peak#	Ret. Time	Area	Height	Area%
1	13.867	16070	2620	0.480
2	14.408	44211	5556	1.322
3	15.517	8728	1296	0.261
4	15.817	3197366	362441	95.587
5	16.108	34420	3038	1.029
6	17.950	44171	5649	1.321
Total		3344966	380601	100.000

Figure S2. The HPLC report of MDP1.

Summary statistics

All-Atom Contacts	Clashscore, all atoms:	0		100th percentile* (N=1784, all resolutions)
	Clashscore is the number of serious steric overlaps (> 0.4 Å) per 1000 atoms.			
Protein Geometry	Poor rotamers	1	5.56%	Goal: <0.3%
	Favored rotamers	17	94.44%	Goal: >98%
	Ramachandran outliers	0	0.00%	Goal: <0.05%
	Ramachandran favored	20	95.24%	Goal: >98%
	Rama distribution Z-score	-1.50 ± 1.57		Goal: abs(Z score) < 2
	MolProbity score^	1.40		97th percentile* (N=27675, 0Å - 99Å)
	Cβ deviations >0.25Å	0	0.00%	Goal: 0
	Bad bonds:	0 / 172	0.00%	Goal: 0%
	Bad angles:	2 / 229	0.87%	Goal: <0.1%
Peptide Omegas	Cis Prolines:	0 / 1	0.00%	Expected: ≤1 per chain, or ≤5%
	Twisted Peptides:	1 / 22	4.55%	Goal: 0
Low-resolution Criteria	CaBLAM outliers	1	5.3%	Goal: <1.0%
	CA Geometry outliers	0	0.00%	Goal: <0.5%
Additional validations	Chiral volume outliers	0/30		
	Waters with clashes	0/0	0.00%	See UnDowser table for details

In the two column results, the left column gives the raw count, right column gives the percentage.

* 100th percentile is the best among structures of comparable resolution; 0th percentile is the worst. For clashscore the comparative set of structures was selected in 2004, for MolProbity score in 2006.

^ MolProbity score combines the clashscore, rotamer, and Ramachandran evaluations into a single score, normalized to be on the same scale as X-ray resolution.

Figure S3. Analysis output: all atom contacts and geometry for MDP1.

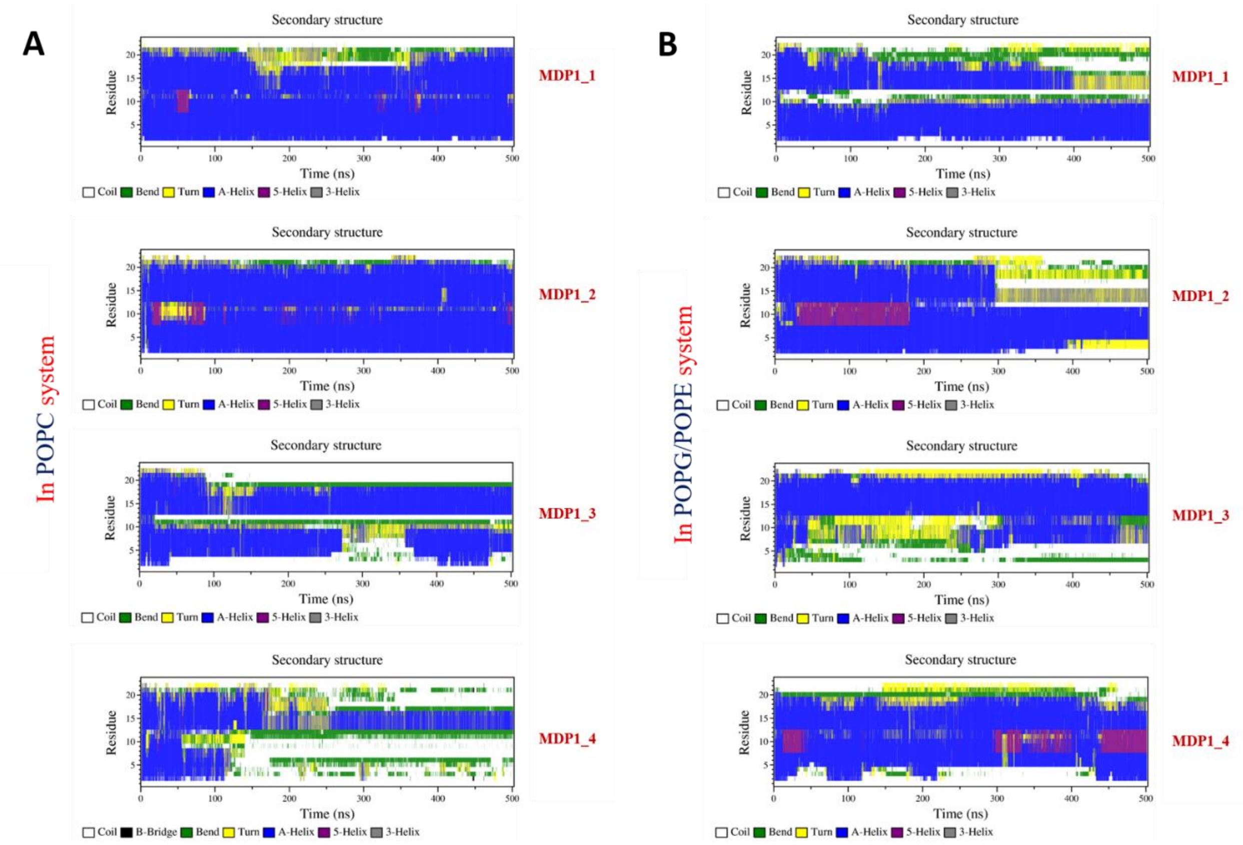

Figure S4. Secondary structures of peptides in POPC (A) and POPG/POPE (B) simulated systems.
